# Assessment of Influenza D Virus in Domestic Pigs and Wild Boars in France: Apparent Limited Spread within Swine Populations Despite Serological Evidence of Breeding Sow Exposure

**DOI:** 10.3390/v12010025

**Published:** 2019-12-24

**Authors:** Stéphane Gorin, Christelle Fablet, Stéphane Quéguiner, Nicolas Barbier, Frédéric Paboeuf, Séverine Hervé, Nicolas Rose, Gaëlle Simon

**Affiliations:** 1 Swine Virology Immunology Unit, Ploufragan-Plouzané-Niort Laboratory, French Agency for Food, Environmental and Occupational Health and Safety (ANSES), 22440 Ploufragan, France; stephane.gorin@anses.fr (S.G.); stephane.queguiner@anses.fr (S.Q.); nicolas.barbier@anses.fr (N.B.); severine.herve@anses.fr (S.H.); 2 Epidemiology, Health and Welfare Unit, Ploufragan-Plouzané-Niort Laboratory, French Agency for Food, Environmental and Occupational Health and Safety (ANSES), 22440 Ploufragan, France; christelle.fablet@anses.fr (C.F.); nicolas.rose@anses.fr (N.R.); 3 SPF Pig Production and Experimentation, Ploufragan-Plouzané-Niort Laboratory, French Agency for Food, Environmental and Occupational Health and Safety (ANSES), 22440 Ploufragan, France; frederic.paboeuf@anses.fr

**Keywords:** influenza D virus, swine, pig, wild boar, epidemiology, serology, PB1-gene RT-qPCR, porcine respiratory disease complex, influenza-like illness

## Abstract

In order to assess influenza D virus (IDV) infections in swine in France, reference reagents were produced in specific pathogen free pigs to ensure serological and virological analyses. Hemagglutination inhibition (HI) assays were carried out on 2090 domestic pig sera collected in 2012–2018 in 102 farms. Only 31 sera from breeding sows sampled in 2014–2015 in six farrow-to-finish herds with respiratory disorders contained IDV-specific antibodies. In two of them, within-herd percentage of positive samples (73.3% and 13.3%, respectively) and HI titers (20–160) suggested IDV infections, but virus persistence was not confirmed following new sampling in 2017. All growing pigs tested seronegative, whatever their age and the sampling year. Moreover, PB1-gene RT-qPCR performed on 452 nasal swabs taken in 2015–2018 on pigs with acute respiratory syndrome (137 farms) gave negative results. In *Corse*, a Mediterranean island where pigs are mainly bred free-range, 2.3% of sera (*n* = 177) sampled on adult pigs in 2013–2014 obtained low HI titers. Finally, 0.5% of sera from wild boars hunted in 2009–2016 (*n* = 644) tested positive with low HI titers. These results provide the first serological evidence that sows were exposed to IDV in France but with a limited spread within the swine population.

## 1. Introduction

Influenza D virus (IDV), the fourth genus of influenza virus among the *Orthomyxoviridae* family, is supposed to circulate mainly in cattle which seems to be the natural reservoir. Since its discovery in 2011 [1], it has been detected in cattle in the USA [2,3,4], France [5], Italy [6,7], Luxembourg [8], Japan [9,10], China [11], Ireland [12,13], United Kingdom [14] as well as in small ruminants in the USA [15], China [11], Ireland [13], Togo and Benin [16]. However, little is still known about exact IDV host range. As suggested by virus detections, serological investigations and/or experimental infections, other animal species may be susceptible to IDV, among which pigs [1], feral swine [17], horses [18], camels [16], guinea pigs [19] and ferrets [1]. Furthermore, anti-IDV antibodies have been detected in humans, with a higher prevalence among cattle workers as compared to the general population, highlighting a potential occupational zoonotic risk [20].

Questions about IDV host range relate especially to the role pigs may play in IDV ecology. Indeed, the virus was first isolated from a pig exhibiting a respiratory syndrome [1]; pigs were demonstrated to be sensitive to IDV infection experimentally [1,21], and similarly to cattle, IDV was isolated or molecularly detected in pigs in different continents, i.e., America [1], Asia [11] and Europe [6,7,8,22]. Serological investigations also confirmed IDV infections in domestic pig and feral swine populations, although with very different positivity rates from one country and/or one study to another [1,7,8,13,17]. 

In France, IDV was isolated from cattle [5], and a recent large-scale study has demonstrated a seroprevalence of 47.2% in cattle and 1.5% in small ruminants [23]. Since IDV is present on the territory, other animal species could harbor the virus. Thus, in order to provide additional information about IDV infections in swine, we investigated IDV circulation among domestic pigs and wild boars in France. Reference reagents were first produced on specific pathogen free pigs to ensure the accuracy of the serological and virological assays in swine. Analyses were performed on archived samples taken from domestic pigs with acute respiratory syndromes for most of them, or from hunted wild boars.

## 2. Materials and Methods

### 2.1. Samples

**Figure 1:**
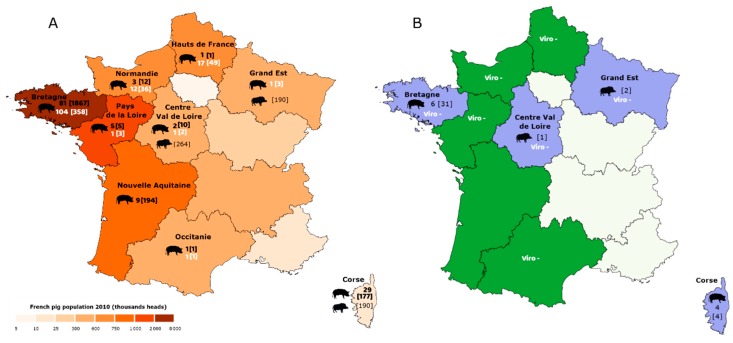
Geographical location of domestic pig and wild boar samples that were analyzed (**A**) and results of serological and virological analyses (**B**) for influenza D virus (IDV) infection in metropolitan France. The lines delimitate the administrative regions, whose names are given when appropriate. (**A**) The colors indicate the pig population size in each administrative region according to the legend provided on the map, based on data provided by the National Agricultural Census [24]. The numbers of pig farms that were tested serologically are given in black color (bold font) close to a pig picture where appropriate, with the number of tested sera in brackets. The numbers of pig farms that were tested for IDV genome are given in white color (bold font), with the numbers of tested nasal swabs in brackets. The numbers of tested sera from wild boars are given in black color in brackets close to a wild boar picture where appropriate. (**B**) The administrative regions where sera tested positive are colored in purple; those where all the tested sera were found negative are colored in green; the regions that were not tested are colored in white. The numbers of positive pig farms are indicated close to a pig picture, with the number of positive sera in brackets. The numbers of positive sera from wild boars are indicated in brackets close a wild boar picture. The results of IDV genome detection, all negative, are indicated as “Viro –“.

First, 2267 sera from domestic pigs issued from 131 farms were selected from six archived banks constituted from 2012 to 2018 by the National Reference Laboratory (NRL) for Swine Influenza or the Epidemiology, health and welfare unit, ANSES, Ploufragan, France. Most sera (*n* = 1867; 81 farms) were sampled in *Bretagne*, the highest pig populated administrative region of France (Figure 1A). They originated from three banks. Bank #1 comprised 1048 sera obtained from breeding sows sampled between January 2014 and June 2015 in 35 farrow-to-finish herds (30 sows/herd) with respiratory disorders. Bank #2 comprised 488 sera taken in November 2013–February 2014 in 10 herds with respiratory disorders, from 16 or 22 week-old pigs (15 pigs/batch). Bank#3 comprised 331 sera obtained between 2012 and 2018 in 36 farrow-to-finish herds, from growing pigs of different physiological stages (from nursery to slaughtering). Other sera were taken from growing pigs reared in *Nouvelle Aquitaine* and *Occitanie*, two regions in the Southwestern part of France (bank #4; *n* = 195; 10 farms; 2013–2018), among which 120 were free-range growing pigs (1 farm), and from *Centre Val de Loire*, *Pays de la Loire*, *Normandie* and *Hauts de France*, located in the Center and Northern part of the country (bank #5; *n* = 28; 11 farms; 2012–2018) (Figure 1A). Finally, sera obtained at slaughter from free-range adult pigs (1.5–3 years of age) reared in *Corse*, an island in the north-western Mediterranean Sea (15 km North of Sardinia and 90 km West of Tuscany in Italy), were included in the study (bank #6; *n* = 177; 29 farms; 2013–2014) (Figure 1A).

An additional bank (bank #7) was constituted in March 2017 following a specific new sampling plan in two of the farrow-to-finish herds where sows were previously sampled in bank #1. Bank #7 comprised 45 sera from sows (15 and 30/herd, respectively) and 60 sera from growing pigs (30/herd).

Sera from wild boars were selected within archived banks from the NRL for Classical Swine Fever and Aujeszky’s Disease, Ploufragan, France. These sera originated from three different regions, *Grand Est* (*n* = 190, 2012–2016), *Centre Val de Loire* (*n* = 264, 2014–2018) and *Corse* (*n* = 190, 2009–2015) (Figure 1A).

Nasal swabs taken on domestic pigs were randomly selected within the collections of the NRL for Swine Influenza, Ploufragan, France. They were all (*n* = 452; 137 farms) obtained thanks to an event-driven surveillance conducted in 2015–2018 in herds with acute respiratory syndrome. All these nasal swab supernatants previously tested negative for influenza A virus using M-gene RT-qPCR [25]. These samples originated from pigs reared in *Bretagne* (*n* = 358; 104 farms), *Normandie* (*n* = 36; 12 farms), *Hauts-de-France* (*n* = 49; 17 farms), *Pays de la Loire* (*n* = 3; 1 farm), *Grand Est* (*n* = 3; 1 farm), *Centre Val de Loire* (*n* = 2; 1 farm) and *Occitanie* (*n* = 1; 1 farm) (Figure 1A).

### 2.2. Virus Strains

Virus strain D/Bovine/Nebraska/9-5/2012 was kindly provided by B. Hause, Kansas State University, USA, and M. Ducatez, UMR IHAP, Toulouse, France. It was propagated for 6 days on 24 h layer of swine testicular cells (ATCC, Manassas, VA, USA) cultured using Dulbecco’s modified Eagle’s medium (DMEM, L0064-500) (Biowest, Nuaillé, France) supplemented with 1% penicillin-streptomycin (L0018-100) (Dominique Dutscher, Brumath, France) and 1 µg/mL of tolysulfonyl phenylalanyl chloromethyl ketone treated trypsin (TPCK, LS003740) (Whortington biochemical corporation, Lakewood, NJ, USA) and incubated at 37 °C with 5% CO_2_. Virus growth was checked by PB1-gene RT-qPCR as described hereafter. In addition, haemagglutination (HA) activity was tested using 0.5% chicken erythrocytes following standard protocols for swine influenza A virus (swIAV) [26]. The virus was further propagated for 6 days (second passage) on ST cells for inoculum production. Virus titer was determined by inoculating ST cells with 100 µL of 10-fold serial dilutions of virus stock. After 6 days of incubation, HA test was carried out to ascertain unclear cytopathic effect, and the 50% tissue culture infectious dose (TCID_50_) was calculated by the Reed-Muench method considering as positive all dilution points giving a HA test result greater than or equal to 2.

Strains representative for enzootic lineages of swIAV circulating in pigs in France since 2009 [27,28] were provided by the NRL for Swine Influenza: A/Swine/CôtesdArmor/0388/2009 (IAV-H1_av_N1), A/Swine/Sarthe/0255/2010 (IAV-H1N1pdm), A/Swine/France/57-140136/2014 (IAV-H1N1pdm), A/Swine/Scotland/410440/1994 (IAV-H1_hu_N2) and A/Swine/Flandres/1/1998 (H3N2). Virus strains B/Brisbane/60/2008 (Victoria lineage) (IBV-V) and C/Johannesburg/1/1966 (ICV) were provided by S. van der Werf, National Reference Center for Respiratory Viruses, Institut Pasteur, Paris, France. Strain B/Massachussets/02/2012 (Yamagata lineage) (IBV-Y) was provided by M. Rosa-Calatrava, VirPath Laboratory, Lyon, France. They were all propagated on embryonated chicken’s eggs and titrated following standard protocols [26].

### 2.3. Production of Reference Reagents in Specific Pathogen Free Pigs

Three 8-week-old specific pathogen free (SPF) pigs were inoculated by the tracheal route with 10^4^.^5^ TCID_50_ of D/Bovine/Nebraska/9-5/2012. These animals were obtained from the experimental pig herd of the French Agency for Food, Environmental and Occupational Health and Safety (ANSES) at Ploufragan, France, which holds an agreement for animal experimentation delivered by the *Direction Départementale de la Protection des Populations des Côtes d’Armor* (Departmental Directorate for Protection of the Populations) (registration number C-22-745-1). The pigs were known to be free from swIAV. The experiment was performed in biosecurity level 3 facilities. It was approved by the French national committee for ethics in animal experimentation ANSES/ENVA/UPEC and authorized by the French Ministry for Research (approval N° 10/11/15-6). Nasal swabs were taken daily until day 14 post-inoculation (pi), and virus shedding was followed in nasal swab supernatants by PB1-gene RT-qPCR as described below. Swine IDV isolation was attempted from PB1-gene positive nasal swabs by propagation on ST cells and was successful for one sample taken at day 13 pi. Thus, D/Swine/France/150445/2015 isolate was further propagated and titrated on ST cells to provide a swine IDV reference antigen for diagnosis analyses. On day 21 pi, the three pigs were injected intramuscularly with the same dose of D/Bovine/Nebraska/9-5/2012 as for first inoculation, mixed (*v*/*v*, final volume 3 mL) with Montanide ISA 206 adjuvant (Seppic, La Garenne-Colombes, France). Blood samples were taken every week after the initial challenge for seroconversion monitoring until day 35 pi when pigs were euthanized, and large volumes of hyper-immune sera were collected. Hyper-immune sera directed against each of the four swIAV, the ICV and the two IBV reference strains were previously produced in SPF pigs following the same procedure under (approval N° 12/07/16-1).

Finally, 174 sera were obtained from not inoculated SPF pigs and used as negative controls. They were from animals of different ages and physiological stages, distributed as follows: 20 piglets from farrowing (1–2 weeks of age), 66 piglets from nursery (7–9 weeks of age), 16 fattening pigs (4–6 months of age) and 72 adult breeding pigs (7–33 months of age).

### 2.4. Hemagglutination Inhibition Assay for the Detection of Anti-IDV Antibodies in Swine Serum

Hemagglutination inhibition (HI) assay for the detection of anti-IDV antibodies in swine serum was performed in accordance with the standard protocols applied for the detection of antibodies directed against swIAV [26]. Briefly, sera were treated with receptor destroying enzyme (Cholera filtrate, C8772) (Merck, Darmstadt, Germany) diluted 1:5 overnight at 37 °C, followed by heat inactivation for 30 min at 56 °C and adsorption on chicken’s erythrocytes at 4 °C under stirring. Two-fold serial dilutions of sera were carried out in U-bottom 96 plates, then 4 HA units of D/Swine/150445/2015 and 0.5% chicken’s erythrocytes were sequentially added following an incubation time of 35 min for each step. HI titer was determined by the highest dilution for which the packed red blood cells were teardrop-shaped after tilting the plate for 45° for 30 s.

### 2.5. Duplex PB1-Gene/β-Actin RT-qPCR

Viral RNA was extracted from cell culture or nasal swab supernatants using Macherey Nagel Nucleospin RNA^©^ or Macherey Nagel Nucleospin 8RNA^©^ (Macherey Nagel, Hoerdt, France) according to manufacturer’s instructions. Detections of IDV genome together with swine host genome were carried out using *PB1* target gene specific primers and probe [1] and porcine *β-actin* reference gene primers and probe [29], respectively. The duplex RT-qPCR was adapted using GoTaq^®^ Probe 1-Step RT-qPCR System (Promega, Madison, WI, USA) under a 25µl volume and run on a MX3005P qPCR System (Agilent Technologies, Santa Clara, CA, USA). Reverse transcription (RT) step was performed for 30 min at 45 °C and, after an inactivation step of 2 min at 94 °C, there were 40 cycles of 10 s at 94 °C and 30 s at 60 °C. Fluorescence data were collected at the end of each of the 40 cycles. 

## 3. Results

### 3.1. Specificity of Hemagglutination Inhibition Assay for Detection of Anti-IDV Antibodies in Swine Serum

The three SPF pigs inoculated with D/Bovine/Nebraska/9-5/2012 exhibited specific anti-IDV antibodies in sera two weeks after the initial challenge and/or after the adjuvant booster (data not shown). One hyper-immune serum was selected as a positive control for cross-HI tests and further analyses of unknown swine sera. This serum obtained HI titer of 640–1280 when tested with D/Swine/France/150445/2015, while hyper-immune sera against swIAV, IBV and ICV reference strains obtained homologous HI titers ranging from 320 to 2560 (Table 1).

No cross-reaction was observed between D/Swine/France/150445/2015 and antibodies directed against the different swIAVs known to circulate in pigs in France, whatever their lineage (Table 1). Similarly, the swine IDV antigen did not show any reaction with antibodies directed against IBV or ICV reference strains. Reciprocally, the swine IDV hyper-immune serum did not show any reaction with any of the swIAV, IBV and ICV reference strains (Table 1). The 174 sera taken from non-inoculated SPF pigs did not give any HI titer when tested against D/Swine/France/150445/2015 except one that exhibited a slight background noise, with a HI titer of 10.

In light of these results, the swine IDV HI test, using D/Swine/France/150445/2015 as an antigen and the swine IDV hyper-immune serum as a positive control, was estimated to be strongly specific for the detection of anti-IDV antibodies in swine sera. The positive threshold titer was set at 20.

### 3.2. Screening of Domestic Pig Sera for Antibodies Directed against Influenza D Virus

Swine IDV HI tests were first performed on 2267 domestic pig sera collected in 131 farms from 2012 to 2018 (banks #1–6) (Figure 1A). We detected 35 (1.5%) positive sera originating from 10 (7.6%) herds located either in *Bretagne* or in *Corse* (Figure 1B). Six positive herds (named A–F) were located in *Bretagne,* and the animals were sows (from bank #1) sampled in 2014–2015 (Table 2). Farms A, C and D had only 1/30 and farm B only 2/30 positive sera, all with HI titers of 20. By contrast, farms E and F exhibited higher positive rates, with 22/30 (73.3%; HI titers 20–160) and 4/30 (13.3%; HI titers 20–80) positive sera, respectively. The four other positive farms (named G–J) were situated in the northern part of *Corse* and the sampled animals (from bank #6) were adult domestic pigs (*n* = 12, 6, 14 and 7, respectively) (Table 2). In each of these four farms, only one serum tested positive with HI titers of 20–40. 

None of the growing pigs sampled in intensive herds located in *Bretagne* (banks #2–3) or other regions in continental France (banks #4–5) were detected seropositive.

In order to evaluate the possible persistence and/or recurrence of IDV infections in the two herds that obtained the highest numbers of positive samples as well as the highest HI titers, i.e., farms E and F located in *Bretagne*, new samples were taken in March 2017, both in sows and growing pigs (bank #7) (Table 3). In farm E, 2/15 sows tested positive with HI titers of 20 and 80, respectively. In farm F, only 1/30 sow exhibited an HI titer of 20. The 30 fattening pigs sampled in each herd were seronegative (Table 3).

Thus, ultimately, a total number of 2372 serum samples from domestic pigs were tested in swine IDV HI test, among which 38 tested positive, leading to a global 1.6% (95% CI: 1.1–2.1) positive rate at the animal level.

### 3.3. Screening of Wild Boar Sera for Antibodies Directed against Influenza D Virus

IDV circulation in wild boars was investigated by analyzing 644 sera originating from three different regions (Figure 1A). Only three sera tested positive (0.5%) (Figure 1B). Two of them were obtained in *Grand Est*, in December 2015 and January 2016, respectively, with mean HI titers of 30 (two independent assays). The third one was obtained in *Centre Val de Loire* in November 2016, and its mean HI titer was 33 (three independent assays). No anti-IDV antibodies were detected in wild boar sera from *Corse*.

### 3.4. Screening of Pig Nasal Swab Supernatants for Influenza D Virus Genome

The 452 nasal swabs sampled in 137 pig herds exhibiting a respiratory outbreak and located all around continental France tested negative with PB1-gene RT-qPCR (Figure 1A,B). It has to be noted that one of these herds that experienced an outbreak in September 2017 was farm F, which tested positive for anti-IDV antibodies in May 2015 and March 2017. Thus, no virological proof of IDV circulation in this farm was established during this clinical event.

## 4. Discussion

In this study, we provided the first serological evidence that some breeding sows have been exposed to IDV in France. Within 35 tested farrow-to-finish herds located in *Bretagne*, the highest pig populated area, sows tested positive in 17.2% of them. The 2.9% positive sera (*n* = 1048) were sampled in 2014–2015, indicating that sows would have been infected during this period of time, or shortly before. The within-herd percentage of positive samples ranged from 3.3% to 73.3%, with HI titers up to 160. Such a high within-herd percentage of positive results does not leave any doubt about a viral passage. However, IDV circulation did not seem to have been maintained in these sow herds, as revealed by analyses performed on new sera sampled in 2017 in two of them. Indeed, two-three years after initial detection of positive sera, only a few sows were still seropositive, with reduced HI titers, which would indicate that the last IDV infection may have occurred quite a long time ago. Moreover, the residual antibodies detected in March 2017 could be assigned to the same viral episodes than those evidenced after the first sampling plan, because it cannot be excluded that some of the youngest sows sampled in May 2015 in herd F could have been sampled a second time, 22 months later, in March 2017.

Whereas IDV circulation among cattle and small ruminants was also serologically evidenced in *Bretagne* [23], further investigations would be necessary to know if IDV infections both in swine and cattle in this region were related or not. In our study, only one positive sow herd was located close (less than 100 m far away) to a dairy cattle farm while IDV transmission from infected calves to sentinel calves through aerosols was only demonstrated for a short distance of three meters under a directed airflow [30].

Surprisingly, none (0%) of the tested growing or finishing pigs (*n* = 1102) were found seropositive towards IDV, whatever their location in continental France, their age and the period of time they were sampled. Among them, 120 were pigs reared in free-range herds located in *Nouvelle-Aquitaine* in the Southwestern part of France, some of which were mixed pig and cattle farms; thus, we were wondering if pigs could have been exposed to IDV at a higher risk in these farms, but these samples also tested negative. For the moment, no data is available about IDV seroprevalence in cattle in this region, but as bovine and small ruminants were detected seropositive in *Pays de la Loire* and *Occitanie,* two neighbor regions, once can suppose IDV would be also circulating in cattle in *Nouvelle-Aquitaine* [23].

By contrast, we detected 2.3% seropositive adult pigs in 13.8% of tested herds located in *Corse.* In this mountainous and forested island, livestock farming (sheep, goats, pigs and cattle) is an important economic activity, and most animals, including pigs, are traditionally bred free-range [31]. Thus, pigs could have been contaminated by direct or indirect contact with domestic bovine animals or small ruminants; however, no data is available about IDV in cattle there to sustain this hypothesis. Pig infections following contact with infected wild boars would be less likely as in this study none of the tested Corsican wild boars were seropositive for IDV.

Overall, even including results from *Corse* where pig breeding is quite different from that in continental France, the seropositivity rate was only 1.6% at the animal level (*n* = 2372), which suggests that IDV would be much less present in domestic pigs in France than in Italy (11.7%) [7], USA (9.5%) [1], Luxembourg (5.9%) [8] or Ireland (5.6%) [13].

Moreover, the IDV genome was not detected in any of the tested 452 nasal swabs (137 herds), even though they were from pigs who exhibited a respiratory outbreak but were not IAV infected. In Northern Italy, where the seroprevalence was reported to be higher, the virus genome was detected thanks to passive surveillance in several regions. In a first study, IDV genome was detected from 1 sow (*n*= 150) sampled in 2015 [6], and in a second one, 2.3% of tested samples (*n* = 845) were found positive and were issued from 9 herds (2%) sampled in 2015–2016 [7]. However, in Luxembourg, where the seroprevalence was lower, the IDV genome detection in nasal swabs was also a very rare event, as only 3/427 (0.7%) nasal swabs sampled in 2014–2015 gave an amplification curve in PB1-gene RT-qPCR [8]. Thus, the very low seroprevalence of IDV in domestic pigs in France (this study) and the short shedding period reported after experimental infection [1] probably contributed to the absence of detection of IDV genome in nasal swab samples in this country. Nevertheless, our virological results are consistent with those obtained in a large-scale study conducted on 3753 nasal swabs taken from pigs with respiratory illness, in 682 farms located in twelve European countries, including France but excluding Italy and Luxembourg [22]. Among them, 1166 nasal swabs were sampled in 2015–2017 on pigs reared in France, and they all tested negative towards IDV in a tetraplex RT-qPCR. Only one nasal swab taken in a German herd was reported to contain IDV genome.

One may wonder if virological investigations should be conducted randomly better than only focusing on pigs with respiratory illness. Indeed, IDV infections may possibly occur without inducing compelling clinical signs, as virological investigations conducted in China on asymptomatic pigs led to a 2% detection rate [11]. However, in this country, the detection rate increased until reaching 40% of the analyzed samples taken from pigs with severe respiratory syndrome, which is also much higher than that obtained in Italy, making difficult to compare the situations.

At the European level, differences in serological and virological positive rates between France and Italy may relate to the herd types where the samples were obtained, and more globally to herd management practices. In France, most of tested sera came from farrow-to-finish herds, as the three quarters of farms where pigs with clinical respiratory signs are sampled for diagnosis analyses are from that herd type [32]. By contrast, in Italy, the herds included in the assessment of IDV infections were only finishing farms and the sampled pigs were older [7]. In this type of livestock management, several batches of piglets from different breeding farms may be mixed together promoting pathogen’s transmission, possibly including IDV. In addition, late slaughtering may increase the infection’s risk at the animal level. Moreover, it has to be noted that IDV seroprevalence in cattle was also lower in France than in Luxembourg [8] and Ireland [12,13]. As cattle is considered a reservoir for IDV, the lower prevalence in this species in France would also partially explain the lower serological rate and the absence of virus detection in pig herds.

Given that a previous study demonstrated anti-IDV antibodies in a high proportion of feral swine sera sampled in 2012–2013 in the USA and confirmed these animals were susceptible to the virus experimentally [17], it could be hypothesized that wild boars may play a role in its transmission to domestic species. In this study, we tested sera from wild boars hunted in France in 2013–2014, and three (0.5%) were found positive, indicating that sporadic infectious events might have occurred. However, they would be even rarer than IAV infections [31,33], suggesting that wild boar would not play a significant role in IDV ecology.

In conclusion, this study provided a first serological evidence that sows have been exposed to IDV in France, but the IDV spread in the swine population may be very limited. However, it will be informative to continue monitoring and remain vigilant with regard to this pathogen, which under certain conditions could perhaps play a role in the porcine respiratory disease complex as suggested by others [1,7].

## Figures and Tables

**Table 1:** Cross-hemagglutination inhibition (HI) assays between hyper-immune sera produced in SPF pigs and reference antigens from D, A, B and C types. Titer ranges were obtained from at least four assays conducted during two independent experiments. Homologous titers are indicated in bold font.

Antigen *	Swine Hyperimmune Sera Containing Antibodies Directed against Reference Influenza Virus Strains *							
	IDV	IAV-H1_av_N1	IAV-H1N1p ^$^	IAV-H1_hu_N2	IAV-H3N2	IBV-V	IBV-Y	ICV
IDV	**640–1280**	<10	<10	<10	<10	<10	<10	<10
IAV-H1_av_N1	<10	**640–1280**	<10	<10	<10	<10	<10	<10
IAV-H1N1p ^$^	<10	<10–10	**640–1280**	10–20	<10	<10	<10	<10
IAV-H1_hu_N2	<10	<10–10	10	**1280–2560**	10–20	<10	<10	<10
IAV-H3N2	<10	<10	<10–10	10–20	**2560–5120**	<10	<10	<10
IBV-V	<10	<10	<10	<10	<10	**640–1280**	10–20	<10
IBV-Y	<10	<10	<10	<10	<10	10	**320–640**	<10
ICV	<10	<10	<10	<10	<10	<10	<10	**1280**

* Virus strains used as reference antigens in HI tests and/or as reference strains for hyperimmune sera production in pigs are given in the Material and Methods section. ^$^ H1N1p = H1N1pdm.

**Table 2:** Origins of domestic pig sera that gave positive results in swine influenza D virus hemaglutination inhibition (HI) tests.

Region	Herd ID *	Sampling Period	Animal Type	Number of Tested Samples	Number of Positive Samples	Positive Rate (%)	HI Titer Range (Mean HI Titer)
*Bretagne*	A	February 2014	sows	30	1	3.3	20 (20)
	B	April 2014		30	2	6.7	20 (20)
	C	April 2014		30	1	3.3	20 (20)
	D	July 2014		30	1	3.3	20 (20)
	E	June 2014		30	22	73.3	20–160 (50)
	F	May 2015		30	4	13.3	20–80 (40)
*Corse*	G	January 2014	adult pigs	12	1	8.3	20 (20)
	H	January 2014		6	1	16.7	40 (40)
	I	January 2014		14	1	7.1	20 (20)
	J	December 2013		7	1	14.3	40 (40)

* ID = identification.

**Table 3:** Results of swine influenza D virus hemagglutination inhibition (HI) tests performed on sera sampled in herds E and F (*Bretagne*) in March 2017.

Herd ID *	Animal Type	Number of Tested Samples	Number of Positive Samples	Positive Rate (%)	HI Titer Range (Mean HI Titer)
E	sows	15	2	13.3	20–80 (40)
	16 w.-o. growing pigs	15	0	0	n.a.
	22 w.-o. growing pigs	15	0	0	n.a.
F	sows	30	1	3.3	20 (20)
	16 w.-o. growing pigs	15	0	0	n.a.
	22 w.-o. growing pigs	15	0	0	n.a.

* ID = identification; w.-o. = week-old; n.a. = not applicable.
